# From animal models to NAMs: a paradigm shift in developmental immunotoxicity testing

**DOI:** 10.1007/s00204-026-04424-w

**Published:** 2026-05-13

**Authors:** Martina Iulini, Véronique de Bruijn, Selma Hurem, Unni C. Nygaard, Saadia Kerdine-Römer, Christiane Spruck, Julia Tigges, Rob Vandebriel, Emanuela Corsini

**Affiliations:** 1 Laboratory of Toxicology and Risk Assessment, Department of Pharmacological and Biomolecular Sciences ‘Rodolfo Paoletti’, Università Degli Studi Di Milano, Via Balzaretti 9, 20133 Milan, Italy; 2 Centre for Health Protection, National Institute of Public Health and the Environment (RIVM), Bilthoven, the Netherlands; 3 Faculty of Veterinary Medicine, Norwegian University of Life Sciences (NMBU), Ås, Norway; 4 Section for Immunology, Norwegian Institute of Public Health, Oslo, Norway; 5 Faculty of Pharmacy, Université Paris-Saclay, Paris, France; 6 Düsseldorf, Germany

**Keywords:** Developmental immunotoxicology (DIT), Immune system maturation, New approach methodologies (NAMs), In vitro models, Organ-on-chip, Physiological maps, Immunotoxicity testing, Risk assessment

## Abstract

Developmental immunotoxicology (DIT) is emerging as a critical area in regulatory toxicology, driven by the recognition that the developing immune system is particularly vulnerable to xenobiotic exposure. Disruptions occurring during fetal or early postnatal life may result in long-lasting alterations in immune competence, tolerance, and disease susceptibility. This review provides a comprehensive overview of immune system development, highlighting key developmental stages from embryogenesis to postnatal maturation and identifying windows of heightened immune system sensitivity to toxicants. By integrating mechanistic insights and methodological advances, this review aims to support the improvement and extension of DIT testing frameworks and the development of predictive tools for regulatory and research applications. Recent advances in New Approach Methodologies offer promising alternatives for modeling human immune ontogeny, while highlighting the challenge of ensuring adequate coverage of critical developmental mechanisms and windows of susceptibility relevant to immunotoxicity. The integration of physiological maps and multi-omics technologies enhances mechanistic understanding, while epidemiological associations between exposures and functional endpoints underscore the real-world relevance of DIT and can identify biomarkers to guide the further development of relevant and sensitive models. Despite these advances, challenges remain, including the scarcity of human reference data, the lack of standardized protocols, and the need for validated test batteries covering diverse mechanisms once the tests have been refined. Addressing these gaps is essential to support the regulatory uptake of DIT data and to advance predictive, mechanistically anchored, and ethically sound strategies for DIT testing.

## Introduction

### Background on immune system development

The human immune system develops across several anatomical sites throughout gestation, and the timing of this process varies by organ system, as also discussed later (see Chapter 2). Immune cells differentiate initially from extra-embryonic yolk sac (YS) progenitors around week 4 of gestation, and subsequently from aorta-gonad-mesonephros (AGM)–derived hematopoietic stem cells (HSCs). The fetal liver and bone marrow (BM), seeded by YS-derived progenitors and AGM-derived HSCs, then take over as the primary sites of hematopoiesis. Immune cells from these primary hematopoietic sites seed developing lymphoid organs such as the thymus, spleen, and lymph nodes (LNs), as well as peripheral non-lymphoid organs (Park et al. 2020).

In mice, the emergence of HSCs and lymphoid and myeloid progenitors is assumed to take place in three developmental windows: 1. Pre-HSCs hematopoiesis: the extra-embryonic YS is a site of early hematopoiesis. Red blood cells develop in the YS before the emergence of HSCs. Myeloid progenitors also arise in the early YS, and their macrophage progeny is maintained in the adult, in the brain, liver, skin, and lungs. The erythro-myeloid progenitors also generate mast cells (MCs), some of which are maintained in adults. Progenitors in the YS can generate αβ and γδ T-cells and B-1, but not B-2, B-cells. 2. Mid-gestation hematopoiesis: multiple hematopoietic populations are present in the mid-gestation fetus. These include HSCs, thymic progenitors, B-1 and B-2 progenitors, and innate lymphoid cell (ILC) progenitors. 3. Adult hematopoiesis: conventional B- and T-cells are produced, while various innate-like lymphocytes are not generated or only to a small extent (Dorshkind & Crooks 2023).

Layering of immune system development (i.e. the various types of lymphocytes that constitute the adult immune system develop in waves from distinct progenitors that emerge at different times during development) also occurs in humans, albeit this is less extensively studied than in mice. Studies of early human hematopoiesis are challenging due to the difficulty of obtaining human fetal tissue, particularly from specific stages of development (Ni et al. 2025). Moreover, lineage tracing models that have been used in mice are unavailable. Thus, in vitro models such as the generation of HSCs and their progenitors from embryonic or induced pluripotent stem cells (iPSCs) are essential (Dorshkind & Crooks 2023). It should be noted, however, that a recent study showed that YS-derived lymphoid progenitors emerge before HSCs in human embryos (Ni et al. 2025).

Single-cell RNA sequencing (scRNA-seq) of fetal, newborn, and adult samples showed that human hematopoietic stem-progenitor cells (HSPC) undergo progressive changes in gene expression throughout the second half of fetal development, resulting in a progressively evolving transcriptional profile at the cell population level (Bunis et al. 2021). Thus, fetal T-cells undergo timed, concerted changes in gene expression that likely govern T-cell function at any given developmental stage. This may suggest that rather than discrete overlapping waves of fetal and adult hematopoiesis, a single HSPC compartment undergoes progressive and continuous changes in gene expression (Burt & McCune 2023).

A landmark study by Suo et al. (2022) used a single-cell atlas of developing human immune cells across prenatal hematopoietic (YS, liver, and BM), lymphoid (thymus, spleen, and LNs), and non-lymphoid peripheral organs (skin, kidney, gut), spanning from week 3 to 16 after gestation. Regarding lymphoid cells, a concerted shift from innate to adaptive immune responses was seen. From week 3 to 9 after gestation, the lymphoid compartment was dominated by natural killer (NK) cells and ILCs, which were supplemented by developing B- and T-cells at 11 weeks after gestation. In addition, the study expands the view of fetal liver and BM as exclusive sites of B-cell lymphopoiesis. While T-cell development is restricted to the thymus, B-cell development is not spatially restricted. This underlines the importance of the thymus for T-cell selection. Another landmark study by He et al. (2025) generated a single-cell atlas of sorted CD45 + cells isolated from various organs, spanning from week 11 to 24 after gestation, and from organs of adults as a control, integrating scRNA-seq and single-cell T-cell receptor (TCR) sequencing. Their observations include: (1) lineage differentiation from thymic progenitor cells to extrathymic precursor cells to either mature naive CD4 + cells and regulatory T-cells (Treg), (2) a more active and widespread T-cell response in second-trimester fetuses than previously assumed, with gradual T-cell activation during development, (3) a higher clonal expansion of memory and activated adult T-cells compared to fetal counterparts, but a similar clonal expansion for Treg, suggesting a role for Treg during the second trimester of pregnancy, (4) a distinct antigen recognition spectrum in fetuses, with higher proportions of TCR α- and β-chains and contraction of the breadth of antigen recognition over time, and (5) the presence of HSCs in various fetal organs, beyond liver, spleen, and BM.

The developing immune system, which forms during embryonic stages, is considered more susceptible than the adult immune system to the effects of xenobiotics, many of which can be immunotoxic, as highlighted in the following chapters. Developmental immunotoxicity (DIT) can result in the alteration of the formation and function of the immune system during critical windows of development and may affect health throughout life. This underscores the need to develop tools to test chemicals with such effects, enabling more effective studies and understanding of DIT. Toxicology is currently undergoing a profound transformation, driven by the dual imperative of ethical responsibility and the need for models that better reflect human biology. Central to this evolution is the adoption of the 3Rs principle (Replacement, Reduction, and Refinement of animal testing) which has catalyzed the development and implementation of New Approach Methodologies (NAMs). These include a growing array of in silico, *in chemico,* and in vitro tools designed to reduce reliance on animal testing while enhancing predictive power (Johnson et al. 2025). By integrating mechanistic insights and methodological advances, this review aims to support the improvement and extension of DIT testing frameworks and the development of predictive tools for regulatory and research applications.

### The importance and challenges of DIT testing data

DIT testing data are essential to protect individuals, especially in the pre- and perinatal phase, from exposure to developmental immunotoxicants by restricting or prohibiting the use of these compounds. Yet, several challenges exist that hamper obtaining sufficient DIT data to draw conclusions and take sufficient mitigation action. Firstly, DIT data are scarce compared with data for most other apical endpoints, as DIT data is requested only in a few cases upon registration of chemicals. The only Organisation for Economic Co-operation and Development (OECD) Test Guideline (TG) that includes DIT testing is the “Extended one generation reproductive toxicity study” (EOGRTS; TG 443) in which effects on lymphocyte subpopulation frequencies (T-cells, B-cells, CD4 + T-cells, CD8 + T-cells, and NK cells) are evaluated, while immune functional endpoints are not assessed. EOGRTS is an information requirement for chemicals manufactured or imported in quantities ≥ 1000 tonnes (in case of equivocal concerns for fertility a EOGRTS can be triggered for a lower quantity). In a limited number of cases, a so-called cohort 3 is included in the EOGRTS. This cohort evaluates exposure effects on the T-cell-dependent antibody response (TDAR). Addition of cohort 3 to the EOGRTS is based on triggers, obtained from (1) screening tests for reproductive and developmental toxicity: TG 421/422, (2) 28- and 90-day oral toxicity studies in adult animals: TG 407/408, or (3) public literature. Under REACH, until 2022 the number of EOGRTS was approximately 380, while the number of cohort 3 studies was around 60. For pharmaceuticals under European Medicines Agency (EMA), until 2023 the number of TDARs was close to 36. About 2/3 of the TDARs were performed to test monoclonal antibodies and thus employed non-human primates. Secondly, from a more general perspective, the breadth and importance of DIT for human health can only be grasped when a possible DIT (and its point-of-departure relative to exposure) has been evaluated for a significant fraction of compounds humans are exposed to. The European Chemicals Agency (ECHA) named DIT a key area in their “Key areas of regulatory challenge” (ECHA, 2024). Third, since developmental immunotoxicants exert their effects through different modes of action (Shao et al. 2013), it is challenging to develop an Integrated Approaches to Testing and Assessment (IATA) based on a limited set of NAMs that together can identify a significant fraction of developmental immunotoxicants. This especially holds in the situation of limited DIT data. While T-cell based NAMs targeting IL-2 signaling, DNA synthesis or T-cell proliferation are promising for identifying compounds that induce DIT via immunosuppressive mechanisms, NAMs addressing alternative modes of action still require further optimization and validation. Promising avenues include in vitro models for T- and B-cell differentiation (Li et al. 2017; Trotman-Grant et al. 2021). Whether these assays may eventually result in a TG awaits further work. Fourth, identification of developmental immunotoxicants would benefit from the possibility of in silico prediction. This requires a sufficient number of positive and negative compounds. Fifth, human DIT data is required to substantiate findings from in vitro and in vivo studies. Over the past years, several epidemiological studies have shown an association of exposure to per- and polyfluoroalkyl substances (PFASs) and reduced vaccination responses in children (Ehrlich et al. 2023). These findings have heightened awareness of the potential health risks posed by PFASs exposure. Importantly, they also provide real-world evidence that DIT is occurring, underscoring the need for improved risk assessment and preventive strategies. See also chapter 3.1 Impact of environmental pollutants, pharmaceuticals, and diet on immune system maturation for further details.

### Relevance of physiological maps in understanding immune maturation

As stated above, human-based NAMs are expected to play a pivotal role in future DIT hazard and risk assessments moving away from animal experiments. A key prerequisite to employ a human NAM-based strategy, however, is a detailed mechanistic understanding of human immune system development and maturation. This is a challenge, since most available knowledge derives from rodent models (Nguyen et al. 2026; Skaggs et al. 2019; Kuper et al. 2016; Vinken et al. 2021). One promising approach to address this gap is the use of physiological maps. Building on the concept of disease maps, which visualize biological mechanisms of various diseases in a standardized and machine-readable format (Ostaszewski et al. 2019), physiological maps focus on representing normal physiological processes (Mazein et al. 2025; Staumont et al. 2025). They provide graphical, interactive depictions of organs (Ladeira et al. 2025) or cell types at the molecular and cellular level, integrating signaling, metabolic and gene regulatory networks (Ostaszewski et al. 2019).

**Fig. 1:**
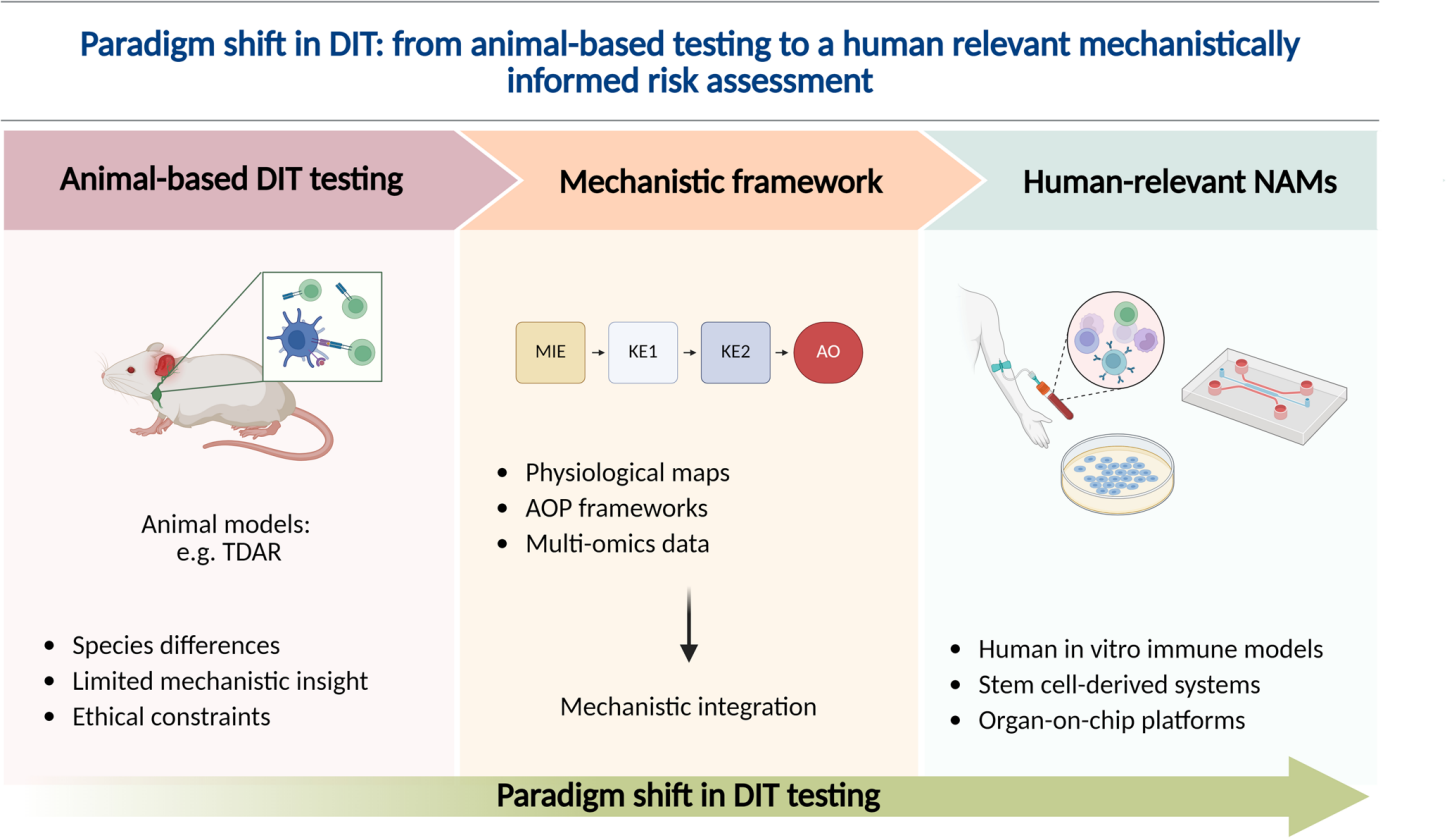
Paradigm shift in DIT. Traditional assessment of DIT has relied largely on animal-based studies, including functional immune assays such as the TDAR. Advances in the mechanistic understanding of human immune development, including physiological maps, AOP frameworks, and multi-omics data, support the development of mechanistically informed approaches. These enable the transition toward human-relevant NAMs, including human in vitro* i*mmune models, stem cell–derived systems, and organ-on-chip platforms, improving the relevance of DIT hazard and risk assessment. Created in BioRender. Iulini, M. (2026) https://BioRender.com/5vrup11

For immune maturation, physiological maps can offer a systematic and comprehensive representation of developmental stages, processes, and organs involved. By capturing the interplay of signaling pathways, metabolic processes, and gene regulation, they allow the visualization of critical windows and mechanisms that underly healthy immune development. Such maps can thus serve both as a reference framework for immunology and as a tool to support the paradigm shift from animal testing towards NAMs in DIT research. They enable the identification of key events and molecular initiating events relevant for building Adverse Outcome Pathways (AOPs), as well as test method development, and help expand these into more integrated AOPs networks (Staumont et al. 2025). By informing the development of relevant AOPs and NAMs, physiological maps help overcome key translational barriers. Thus, the paradigm shift from animal-based to NAM-driven DIT approaches is defined by a move towards mechanistic, human-relevant, and integrative methodologies building on the AOP framework. This transition from traditional animal-based testing toward human-relevant NAMs is illustrated in Fig. 1.

An important step in this direction is the Human Immune System Development Map (HIDmap) (Spruck et al. n.d., *manuscript under revision*), developed by the Leibniz Research Institute for Environmental Medicine (IUF) as the first expert-curated, machine-readable, and publicly accessible representation of prenatal immune system development. Hosted on the Molecular Interaction Network Visualization (MINERVA) platform (Gawron et al. 2016) and using standardized Systems Biology Graphical Notation (Le Novère et al. 2009), the HIDmap integrates published data into organ- and process-specific maturation during in utero development. Within the Partnership for the Assessment of Risk from Chemicals (PARC), the HIDmap is currently being expanded, ensuring broader coverage of immune developmental processes and chemical relevance (Snapkow et al. 2024).

Beyond risk assessment, the physiological maps of human immune system development can identify knowledge gaps, facilitate the detection of immune dysfunctions, and provide a basis for integrating multi-layered data. It further supports the visualization and analysis of experimental datasets, strengthens in vitro and in silico models, and has educational value (Staumont et al. 2025). Ultimately, such physiological maps promote knowledge exchange across immunology, toxicology, regulatory science, and clinical medicine – fostering a deeper understanding of human immune maturation and its practical application in diagnostics, therapy development, and safety evaluation.

## Development of the human immune system

The developing immune system is particularly vulnerable to environmentally induced toxicity compared with the fully mature immune system of adults. Firstly, the dose needed to induce an effect is several orders of magnitude lower. It has also been shown that a broader array of immune parameters is disrupted following exposure to the developing immune system compared to the adult immune system. A disruption of the developing immune system is likely to persist later into life (Dietert et al., 2014). To better understand the plethora of processes that take place during immune system development, immune organogenesis is described in the following section.

### Overview of immune organogenesis

**Fig. 2:**
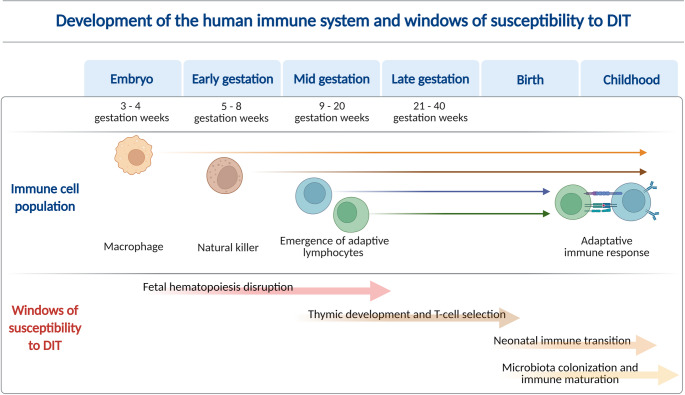
Development of the human immune system and windows of susceptibility to DIT. The human immune system develops progressively during gestation and early life. During gestational weeks 3–4, the YS is the main site of hematopoiesis, followed by the fetal liver, while BM becomes the primary hematopoietic site shortly before birth. Thymus, lymph nodes, and spleen support immune cell differentiation and maturation. Innate immune cells such as macrophages emerge early in development, followed by natural killer cells and, during mid-gestation, the appearance of adaptive lymphocytes. Functional adaptive immune responses become established around birth and continue to mature during childhood. These stages correspond to critical windows of susceptibility to DIT, including fetal hematopoiesis disruption, thymic development and T-cell selection, neonatal immune transition, and postnatal microbiota colonization and immune maturation. Created in BioRender. Iulini, M. (2026) https://BioRender.com/er4w6cb

The human embryo develops from a zygote to a blastocyst during a series of mitotic cleavages. This is followed by key developmental milestones, including gastrulation and organogenesis, which ultimately shape the embryo into a recognizable fetus. In humans, the organogenesis phase extends from approximately the third to the eighth week of gestation. At this stage, the organs are formed in their basic, primitive form and will continue to mature afterwards. Organs such as the liver, BM, thymus, and spleen are pivotal in the maturation of the fetal immune system (Hossain et al. 2022). Several windows of increased susceptibility to DIT have been identified, meaning that exposure during these windows has an especially large impact on the health outcome. An overview of the temporal development of the human immune system and the main windows of susceptibility to developmental immunotoxicity (DIT) is shown in Fig. 2.

### Yolk sac

During the initial stages of gestation (days 16 to 18), the YS is the first site of hematopoietic production. Here, hematopoietic (precursor) cells are formed. Definitive HSCs do not arise within the YS but within the AGM. After 4 weeks of gestation, HSC-like progenitors, NK cell progenitors, erythroid cells, MCs, and ILC progenitors can be observed in the YS (Popescu et al. 2019). The fetal YS also produces primitive macrophages, which subsequently give rise to macrophages in the brain, liver, lung, and epidermis (Gomez Perdiguero et al. 2015; Hoeffel et al. 2015). At around 4 weeks these progenitors migrate to the fetal liver, establishing it as the principal site of hematopoiesis by the fifth to sixth week. The increasing number of nucleated cells between weeks 5 and 10 facilitates the rapid growth of the liver (Hossain et al. 2022).

### Liver

The early liver originates as a diverticulum from the floor of the embryonic gut, initially containing erythrocytes and CD45 + cells that arise from the YS. By the third and fourth weeks of gestation, this rudimentary liver also harbors cells of the monocytic/macrophage lineage, and the developing CD34 + CD45 + population, respectively (Hossain et al. 2022; Kuchma et al. 2015). As fetal development progresses, some intra-aortic hematopoietic clusters and CD34 + CD45 + cells in the fetal liver are replaced by cells derived from the AGM region, coinciding with the emergence of definitive HSCs that give rise to erythroid, myeloid, megakaryocyte, and lymphoid lineages (Slayton et al. 1998). Notably, among the HSC-derived populations, production of monocytes, macrophages, and dendritic cells (DCs) begins, with the earliest DCs identified in the fetal liver by 6 weeks of gestation (Popescu et al. 2019). From 12 weeks onward, various DCs subsets, conventional DCs1 and DCs2, as well as plasmacytoid DCs can be found in different fetal tissues, including the skin, lung, spleen, and thymus (Fogg et al. 2006). Fetal DCs are functionally responsive to Toll-like receptor (TLR) stimulation and play key roles in T-cell differentiation, proliferation, and activation. They also enhance T-cell interleukin 4 (IL-4) production while suppressing T-cell tumor necrosis factor α (TNF-α) release via arginase 2 activity. These functions underscore the importance of DCs in establishing immune tolerance during fetal development (Fogg et al. 2006). Additionally, B-cell lineage cells, including precursors and mature B-cells, first appear in the fetal liver around 7 and 9 weeks of gestation, respectively (Popescu et al. 2019). Thus, the liver serves as the principal site for hematopoietic differentiation and HSCs expansion throughout fetal development until just before birth.

### Bone marrow

BM development is closely associated with the invasion of cartilaginous bone by blood vessels and bone ossification. The formation starts at gestation week 8. The BM grows into the most important source of B-cells (Zakir et al. 2022), with abundant enrichment in the spleen during mid-gestation, and the development of intestinal B-cells occurring from the second trimester until birth. Just before birth, BM takes over as the main hematopoiesis site from the liver (Kuper et al. 2016).

### Thymus

Thymus formation begins at gestational week 6, and by weeks 15–16 the medulla is fully formed. Early lymphoid progenitors migrate from fetal liver to the thymus at week 8 of gestation, where naive T-cells are formed (Haynes & Heinly 1995). These early thymic precursors differentiate into naïve T-cells that then migrate. At gestation week 14, the thymus starts to export single positive CD4 + and CD8 + T-cells to other tissues (Kuper et al. 2016). Treg are generated from naïve T-cells of the fetus, suppressing the proliferation and cytokine secretion of other fetal T-cells (Michaëlsson et al. 2006; Mold et al. 2010). A Treg subset that differentiates perinatally and prevents autoimmunity in mice cannot be replaced by Treg generated in adults (Yang et al. 2015), underlining the developmental layered immune system.

### Lymph node

LN formation is a longer process. During week 5—6 of gestation, the lymph sacs/vessels start to form. Early LN formation, including afferent lymphatics, takes place during gestation week 12—14. During week 25—38 after gestation, a distinct capsule with efferent lymphatics is formed (Kuper et al. 2016).

The LNs begin to form from condensed mesenchymal cells between the 8th and 11th week of gestation, with the capsule formed by the marginal sinus. By the 13th week, the mesenchyme fills the lymphatic sac, and the intermediate sinuses develop, while the first LN structures are found in the cervical and retroperitoneal regions at 8 weeks. The peribronchial, coeliac, mediastinal, axillary, inguinal, pelvic and popliteal LNs can be detected at 9—10 weeks, and the omental and mesenteric LNs at 14 weeks (Hossain et al. 2022). Different lymphoid cell populations including T-cells, B-cells and some innate lymphoid cell populations migrate to the LN and contribute to the generation and shaping of lymphoid organs (Onder & Ludewig 2018).

### Spleen

The spleen begins to form at week 6 after gestation and develops both red and white blood cells during the second trimester. The spleen contributes to T-cell-dependent immunity with the help of DCs. In contrast to the fetal liver, where DCs development has been described, the ontogeny of DCs within the fetal spleen remains largely unexplored (Hossain et al. 2022).

### Key stages of immune system maturation

#### Prenatal immunity

The fetal immune system undergoes programming starting from the first trimester, with innate and adaptive immune cells appearing and multiplying until birth. Monocytes/macrophages are the first innate cells present, followed by granulocytes, NK cells, and B- and T-cell precursors (Hossain et al. 2022). In humans, the maternal and fetal immune systems are in close contact due to the hemochorial nature of the placenta, which is useful to maximize exchange of nutrients and immune factors. The placenta starts to develop early in pregnancy and becomes vascularized between 8 and 12 weeks of gestation. To tolerate the semi allogeneic fetus, the maternal immune system undergoes specific adaptations, and immune cells within the uterine mucosa (decidua) play a key role in regulating trophoblast invasion (Svensson-Arvelund et al. 2014). During prenatal development, fetuses receive maternal immunoglobulin G (IgG) through the placenta, while their immune organs begin to form (Chucri et al. 2010). The placenta also forms a protective barrier against environmental pollutants.

#### Postnatal immunity

At birth, the activation of innate immunity marks a crucial transition as the newborn encounters the external environment with a sharp increase in antigens. Maternal IgGs present in colustrum provide passive protection during the neonatal period while the infant’s immune system matures (Simon et al. 2015). After birth, the newborn must undergo a key immune developmental shift (Th2 to Th1) to establish a productive immune response capable of defending against a wide range of diseases throughout childhood and later life. The dynamic changes and ongoing maturation processes that characterize the developing immune system result in its heightened sensitivity (Dietert & Piepenbrink 2006). There is significant overlap of fetal and postnatally derived hematopoietic precursor cells in the period immediately after birth. The hematopoietic precursor cells influence the quality of immune cells generated and the overall functionality of the immune system. Although newborns can mount a range of pro- and anti-inflammatory responses, their immune system is distinct from the adult immune system, rendering them more susceptible to infections caused by various pathogens (Georgountzou et al. 2017). This also necessitates pediatric vaccination schemes to include maternal vaccinations or multiple immunizations with administrations later in infancy to induce protective responses. In infancy, adaptive immunity emerges as B- and T-cells become more active, and vaccinations stimulate antibody production and induction of antigen-specific T- and B-cells. As children grow from infancy to early childhood, the immune system continues to mature, with increased antibody production and exposure to pathogens strengthening their immunity (Simon et al. 2015). Late childhood and puberty see continued immune maturation and the development of immunological memory from past infections and vaccinations, enhancing the child’s ability to respond effectively to familiar pathogens. An insufficient, excessive or modulated immune response in infants can have consequences, with modern infants being increasingly prone to autoimmune diseases such as type 1 diabetes, allergies, and asthma (Jain 2020).

#### Innate immunity

The innate immune system plays a crucial role in responding to antigens shortly after birth, influencing host defense mechanisms for life. Microbial and commensal microbe recognition is facilitated by pattern recognition receptors on various host cells, essential for regulating inflammation during commensal microbiota establishment and fighting pathogenic viruses and bacteria. Dysregulation of these receptors, particularly in preterm infants, can lead to serious conditions like neonatal sepsis, necrotizing enterocolitis, and impaired viral clearance (Jain 2020). Reduced TLR4 expression on monocytes in preterm infants and those with very low birth weight may increase susceptibility to gram-negative bacterial infections due to the lack of cytokine production (Öster-Waldl et al. 2005; Sadeghi et al. 2007). Proper regulation of innate immune responses in early life is critical for long-term immunity and protection against infections. TLRs expression and distribution are similar in monocytes of newborns and adults, but the functional outcomes of TLRs activation differ in newborns, with preterm infants showing increased IL-10 production and term infants producing more IL-6 and IL-23 (Kollmann et al. 2017). Plasmacytoid DCs (pDCs) play a crucial role in type 1 interferon (IFN) production during viral infections, but exhibit weakened responses in respiratory syncytial virus-infected newborns (Marr et al. 2014). Monocyte-derived DCs and pDCs from umbilical cord blood demonstrate potent IL-12p70 and type 1 IFN production when exposed to certain viruses like cytomegalovirus, human immunodeficiency virus and herpes simplex viruses (Renesson et al. 2009; Zhang et al. 2014, 2013). These findings suggest that TLRs activation on pDCs in response to viral triggers varies between term and preterm infants, despite comparable TLR expression levels.

#### Adaptive immunity

Recent data has challenged the earlier hypothesis of deficient neonatal adaptive immunity, suggesting that neonatal T-cells are a distinct population well-adapted to the perinatal period (Rudd 2020). Neonatal T-cells differ from adult T-cells in origin and functionality, demonstrating a preference for T helper (Th) 2 and Treg differentiation in CD4 + T-cells, and rapid effector production in CD8 + T-cells (Mold et al. 2010; Smith et al. 2018; Wang et al. 2016). Even in the absence of stimulation, neonatal T-cells exhibit distinct gene expression profiles compared to adult T-cells indicating heightened reactivity to antigens (Palin et al. 2013; Smith et al. 2018; Wissink et al. 2015; Yu et al. 2016; Zens et al. 2017). This finding highlights the specialized capabilities of neonatal adaptive immunity, with neonatal T-cells expressing various TLRs such as TLR2 and TLR5, enabling them to respond effectively to microbial substances (Komai-Koma et al. 2004; McCarron et al. 2009; Sinnott et al. 2016). Neonatal T-cells show a predisposition towards tolerance to self and non-self antigens, while being prepared for a rapid immune response when needed. Similarly, neonatal B-cell responses differ from adults, with fetal precursor cells giving rise to B1 B-cells that remain in the spleen for around one-month after birth, producing low-affinity antibodies with broad reactivity (Hardy & Hayakawa 2001). The B-cell population in newborns comprises mainly naïve and transitional or developing B-cells along with a small number of memory cells, illustrating the developmental differences in immune responses during the neonatal period compared to adults.

## Chemicals affecting immune system maturation

Research into DIT has evolved considerably, emphasizing how exposure to a wide array of chemical, pharmacological and nutritional stressors in early life can disrupt the development of the immune system. Such disturbances may lead to changes in immune competence, tolerance, and susceptibility to infection, allergies, or autoimmune diseases later in life (DeWitt et al. 2012). The examination of vulnerable windows of immune development, the mechanistic pathways of disruption and their translation into human-relevant systems is now driving a paradigm shift in DIT hazard assessment and risk evaluation.

### Impact of environmental pollutants, pharmaceuticals, and diet on immune system maturation

The maturation of the immune system is orchestrated through a succession of tightly regulated developmental phases, including HSCs specification and proliferation, thymic seeding and selection, and the establishment of peripheral T- and B-cell repertoires. Each of these stages represents a period of heightened vulnerability during which exposure to foreign substances can induce long-lasting alterations in immune competence, tolerance, and regulation (Dietert 2014). Consequently, exposure to environmental pollutants, pharmacological agents, or nutritional imbalances during gestation, early postnatal life, and infancy requires careful consideration, as these developmental windows are particularly sensitive to disruption. A growing body of evidence demonstrates that insults during these critical periods can shape immune trajectories well into adulthood, influencing susceptibility to infection, allergy, and autoimmune and other immune stimulations (WHO, 2012). The following paragraphs summarize key lines of evidence supporting this concept, while Table 1 provides an overview of human-based data linking early-life exposures to developmental immune alterations across major exposure classes.

**Table 1:** Overview of chemical classes associated with developmental immunotoxicity (DIT). The table lists representative compounds, their predominant immunotoxic mechanisms, and the developmental stages most affected

Exposure category	Substances/agents	Immune targets and developmental endpoints in humans	Evidence type	Key references
Environmental pollutants	Per- and polyfluoroalkyl substance (PFASs)	Reduced vaccine-specific antibody responses (diphtheria, tetanus, rubella, mumps); impaired adaptive immune maturation; increased immune vulnerability during prenatal–early childhood window	Prospective birth cohorts; cross-sectional population studies	Grandjean et al. 2012; Granum et al. 2013; Stein et al. 2016
	Methylmercury; polychlorinated biphenyls	Reduced antibody responses to routine childhood vaccinations; altered cytokine profiles; impaired immune competence	Birth cohorts; epidemiological studies in exposed populations	Bose-O’Reilly et al. 2010; Heilmann et al. 2006
	Fine and ultrafine PM (PM 2.5, PM 0.1)	Epigenetic modification of immune-regulatory genes; impaired Treg development; increased inflammatory immune programming	Human epidemiological and epigenetic studies	Dugershaw et al. 2020; Johnson et al. 2021;
Pharmacological agents	Dexamethasone	Reduced fetal thymus growth; decreased cord-blood T-cell proliferative capacity; enhanced NK-cell cytotoxicity; altered balance between adaptive and innate immunity at birth	Prospective pregnancy cohorts; neonatal immunophenotyping studies	Jones et al. 2020; Kavelaars et al. 1999
	Rituximab, other anti-CD20 mAbs	Transient neonatal B-cell depletion; hypogammaglobulinemia; occasional delayed vaccine responsiveness	Clinical case series; registry-based studies	Klink et al. 2008; Schwake et al. 2024
Nutrition, dietary factors & food contact materials	Deficiency of vitamins A, D, E, zinc	Impaired lymphocyte differentiation; reduced antibody production; compromised mucosal immunity; increased risk of infection and allergy	Human nutritional, observational, and birth-cohort studies	Gombart et al. 2020; Forgie et al. 2020; Mora et al. 2008
	Western-style maternal diet (high fat, refined carbohydrates, low fiber)	Dysregulated neonatal immune programming; pro-inflammatory immune bias; increased susceptibility to metabolic and immune-mediated disease	Observational cohort and translational human evidence	Nelson & Friedman 2024
	Bisphenol A (BPA); phthalates	Skewed Th1/Th2 balance; impaired Treg maturation; increased allergic sensitization in offspring	Human observational studies; regulatory risk assessment	EFSA, 2023

### Environmental pollutants

Persistent environmental chemicals have emerged as key disruptors of immune development, with PFASs representing one of the best-characterized classes of developmental immunotoxicants. Epidemiological evidence from multiple cohorts supports a consistent association between early-life PFASs exposure and impaired vaccine responsiveness in children. In a landmark prospective study from the Faroe Islands, higher prenatal concentrations of perfluorooctane sulfonate (PFOS) and perfluorooctanoic acid were associated with markedly reduced antibody titers against diphtheria and tetanus at ages 5 and 7 years; a doubling of PFOS concentration corresponded to an estimated 39% decline in diphtheria antibody levels at age 5 (Grandjean et al. 2012). These findings were independently confirmed in a Norwegian birth cohort, where higher maternal PFASs concentrations during pregnancy predicted lower antibody levels against rubella and tetanus toxoid in children up to 3 years of age (Granum et al. 2013). Cross-sectional analyses from U.S. adolescents (12–19 years) further reported lower rubella and mumps antibody titers among individuals with elevated serum PFASs levels (Stein et al. 2016). Collectively, these findings identify the prenatal-to-early-childhood window as a critical period for PFAS-induced immune vulnerability and underscore the sensitivity of the developing adaptive immune system to low-level, chronic xenobiotic exposures.

Beyond PFASs, other environmental pollutants, including dioxin-like compounds, heavy metals, and airborne particulate matter (PM), have also been implicated in immune-developmental disturbances. Exposure to the dioxin congener TCDD (2,3,7,8-tetrachlorodibenzo-p-dioxin) during gestation is known to impair thymic development and disrupt T-cell differentiation through activation of the aryl hydrocarbon receptor pathway, resulting in long-lasting suppression of cell-mediated immunity (Jin et al. 2014). Prenatal and early-life exposure to methylmercury and polychlorinated biphenyls has similarly been associated with diminished vaccine responsiveness, increased infections and altered cytokine profiles in both animal models and human cohorts (Bose-O'Reilly et al. 2010; Heilmann et al. 2006; Stølevik et al. 2013). Airborne fine (PM 2.5) and ultrafine (PM 0.1) exposures have also emerged as drivers of DIT. Prenatal or early-life exposure to ambient PM can induce oxidative stress and epigenetic remodeling in critical immune-regulatory genes, thereby impairing Treg development (Johnson et al. 2021). While it is plausible that PM 2.5 may translocate from the respiratory tract to the placenta and fetus (Bové et al. 2019), adverse effects may also occur secondarily to maternal inflammatory responses (Dugershaw et al. 2020; Hougaard et al. 2015). Proposed mechanistic pathways include maternal and placental oxidative stress and inflammation, activation of placental TLRs, impaired growth and hormone secretion, and vascular dysfunction (Dugershaw et al. 2020). Experimental in vivo and in vitro data on micro-and nano-plastic particles also indicate that nanoparticle exposure may perturb macrophage function and antigen presentation, potentiating a chronic pro-inflammatory state (Bianchi et al. 2025).

### Pharmaceuticals

While pharmacologically beneficial, some pharmaceuticals can also exert DIT effects when exposure occurs during gestation or early postnatal life. Pharmaceuticals that cross the placenta or are administered neonatally can interfere with immune ontogeny, resulting in persistent alterations to immune competence and tolerance. Synthetic glucocorticoids, such as dexamethasone, are among the most extensively studied pharmacological agents in DIT. They are administered to pregnant women at risk of preterm delivery. These agents readily cross the placenta and act via glucocorticoid receptors that are expressed in the fetal thymus and HSCs (Cain & Cidlowski 2017; Moisiadis & Matthews 2014). Experimental evidence in rodents indicates that short-term exposure to dexamethasone during pregnancy induces thymic involution, reduces double-positive thymocytes (CD4⁺CD8⁺) and alters the formation of the TCR repertoire (Bakker et al. 1995). These changes persist in early postnatal life, resulting in reduced thymic output and altered T-cell subset ratios. A recent prospective cohort study found that prenatal corticosteroid administration was associated with reduced fetal thymus growth (Jones et al. 2020). These in vivo human observations are complemented by mechanistic in vitro and model-organism studies, which provide further insight into how glucocorticoids act as pharmacological immunotoxicants. For instance, exposure of human cord-blood stem cell-derived DCs precursors to dexamethasone in vitro inhibited their differentiation into immature DCs, suppressed CD86/CD83 expression, increased IL-10 secretion, and reduced T-cell stimulatory capacity (Mainali & Tew 2004). Additionally, a recent zebrafish study revealed that exposure during embryonic stages to concentrations of synthetic glucocorticoids similar to those used therapeutically altered immune cell migration (neutrophils/macrophages) and caused skeletal developmental abnormalities. This extends the concept of glucocorticoid-mediated DIT beyond mammals (Hamilton et al. 2024).

It is also evident that chemotherapeutic and epigenetically active pharmaceuticals can manifest DIT-relevant properties. For instance, administration of cyclophosphamide, an alkylating chemotherapeutic agent, during gestation in mice results in fetal thymic atrophy and cortical lymphocyte depletion (Prakash et al. 2007). Offspring demonstrates decreased thymocyte counts and impaired T-cell responsiveness, thus highlighting a direct teratogenic action on developing lymphoid organs. In utero exposure also impairs reticuloendothelial clearance function in young-adult offspring, indicating durable consequences on immune system homeostasis. In a similar manner, valproic acid (VPA), a histone deacetylase inhibitor, has been demonstrated to exert an effect on immune development through epigenetic mechanisms (Gąssowska-Dobrowolska et al. 2020). In murine models, prenatal VPA exposure has been shown to result in altered cytokine production, increased intestinal inflammation, and microglial activation, thus suggesting an aberrant crosstalk between the developing nervous and immune systems (Gąssowska-Dobrowolska et al. 2020). These findings illustrate how pharmacologically induced epigenetic dysregulation can intersect with DIT pathways by modifying gene networks that are essential for immune differentiation and tolerance.

Monoclonal antibodies (mAbs) and other biologicals that target immune components represent a novel class of DIT-relevant agents. As IgGs cross the placenta efficiently after mid-gestation, it is evident that mAbs administered during pregnancy can reach the fetal circulation (Klink et al. 2008; Schwake et al. 2024). Clinical case studies demonstrate that rituximab and other anti-CD20 mAbs administered in the third trimester cause transient neonatal B-cell depletion and hypogammaglobulinemia, sometimes accompanied by delayed vaccine responsiveness (Klink et al. 2008). Although these effects are reversible within months after birth, they provide evidence that pharmacological modulation of immune pathways during development can have measurable, mechanistically defined immunotoxic outcomes.

### Nutrition and diet

In parallel to these pharmacologically induced perturbations of immune development, nutrition represents a pervasive environmental factor capable of shaping immune maturation throughout early life. Nutrition during the "first 1,000 days" (from conception through to approximately 24 months of life) plays a pivotal role in immune system maturation. This is achieved via two main mechanisms: firstly, directly through nutrient-mediated signaling, and secondly, indirectly via modulation of the gut microbiota and its metabolic products (Fragkou et al. 2021). Adequate levels of micronutrients such as vitamins A, D, E, and zinc are essential for lymphocyte differentiation, antibody production, and maintenance of mucosal barrier function (Gombart et al. 2020; Mora et al. 2008). Conversely, maternal undernutrition or deficiencies in these key nutrients have been associated with impaired thymic development, altered microbial colonization patterns, and increased susceptibility to infection and allergy in offspring (Forgie et al. 2020).

Maternal overnutrition and the consumption of a diet characterized by high fat, refined carbohydrates, and low fiber content have been associated with systemic inflammation and dysregulation of neonatal immune programming (Nelson & Friedman 2024). Maternal high-fat diets promote microbiota dysbiosis, reduce Treg development, and epigenetically reprogram myeloid precursors towards a proinflammatory phenotype in offspring in mice. Such alterations may result in predisposition to metabolic inflammation, asthma, and autoimmune susceptibility later in life. In addition to macronutrient imbalances, chemicals in food contact materials can influence immune development by migrating from the packaging material to the food and subsequent ingestion. Endocrine-active compounds, such as bisphenol A (BPA) and phthalates, which are commonly found in food packaging, have been shown to disrupt the Th1/Th2 balance, inhibit Treg maturation, and enhance allergic sensitization (EFSA, 2023). Therefore, nutrition represents both a risk factor and a protective determinant of immune developmental programming. Optimizing maternal diet quality and minimizing exposure to immunomodulatory food contaminants are essential strategies for supporting immune resilience and reducing long-term disease susceptibility.

Table 1 summarizes the main classes of chemicals known to interfere with immune system maturation, highlighting their primary mechanisms of action, critical windows of susceptibility, and key functional outcomes. The table is intended to provide a quick comparative overview of how different stressors affect distinct components of immune development. Taken together, this body of evidence suggests that chemical exposures during immune ontogeny can lead to immune dysregulation not only through overt cytotoxicity but also via subtle molecular and epigenetic reprogramming of immune developmental trajectories. The persistence of these changes into later life highlights the importance of identifying environmentally sensitive windows of immune vulnerability and integrating mechanistic insights into future DIT testing frameworks.

### Advances in experimental models and in vitro systems

The developing immune system, including the in utero, early-life, and juvenile stages, is generally considered more sensitive to immunotoxicants than the adult immune system (Dietert & Piepenbrink 2006). Lower doses may induce effects compared with adults, and exposures during early life can lead to different and potentially long-lasting alterations in immune function (Luebke et al. 2006; Jusko et al. 2016).

Ideally, each ‘window’ of immune development should be evaluated using dedicated alternative methods, as the distinct immunological waves that occur from fetal life through adolescence may carry different vulnerabilities to immunotoxicant exposure. In practice, however, developing a full suite of assays for every developmental stage is neither feasible nor always necessary. What is essential is the ability to accurately estimate immunologic risk across the entire continuum of immune maturation. Achieving this will require integrative approaches that capture the most sensitive and biologically relevant periods of development, while ensuring that critical pathways of immune competence and tolerance are adequately represented in testing strategies. In the further development of alternative methods, another item to consider is the sex. Sex-specific immunotoxic effects are not universal during early development, nor are they limited to that stage. However, numerous cases show differing immune outcomes between males and females following perinatal exposure to xenobiotics, likely influenced by differences in hormonal environments during immune system development (Hussain et al. 2005). Finally, genetic considerations should be considered. In fact, genetic predisposition to the development of specific endpoints, such as allergy or autoimmunity, is well recognized in both human and animal studies and should be considered during NAM development. Accordingly, model systems should be human-relevant and selected or designed to ensure sufficient genetic susceptibility for the detection of relevant endpoints. As an example, allergy-promoting compounds cannot be adequately evaluated in animal models that are not prone to develop IgE-mediated responses.

### How can we study DIT in vitro?

In vitro assays traditionally used for adult immunotoxicity have been proposed as preliminary screening tools for developmental contexts (OECD 2022). While these assays can provide valuable insights into basic hazard identification, caution is warranted when extrapolating results to early life stages. The developing immune system exhibits heightened sensitivity to developmental immunotoxicants, and standard adult-based thresholds may underestimate risks during critical windows of immune maturation.

**Fig. 3:**
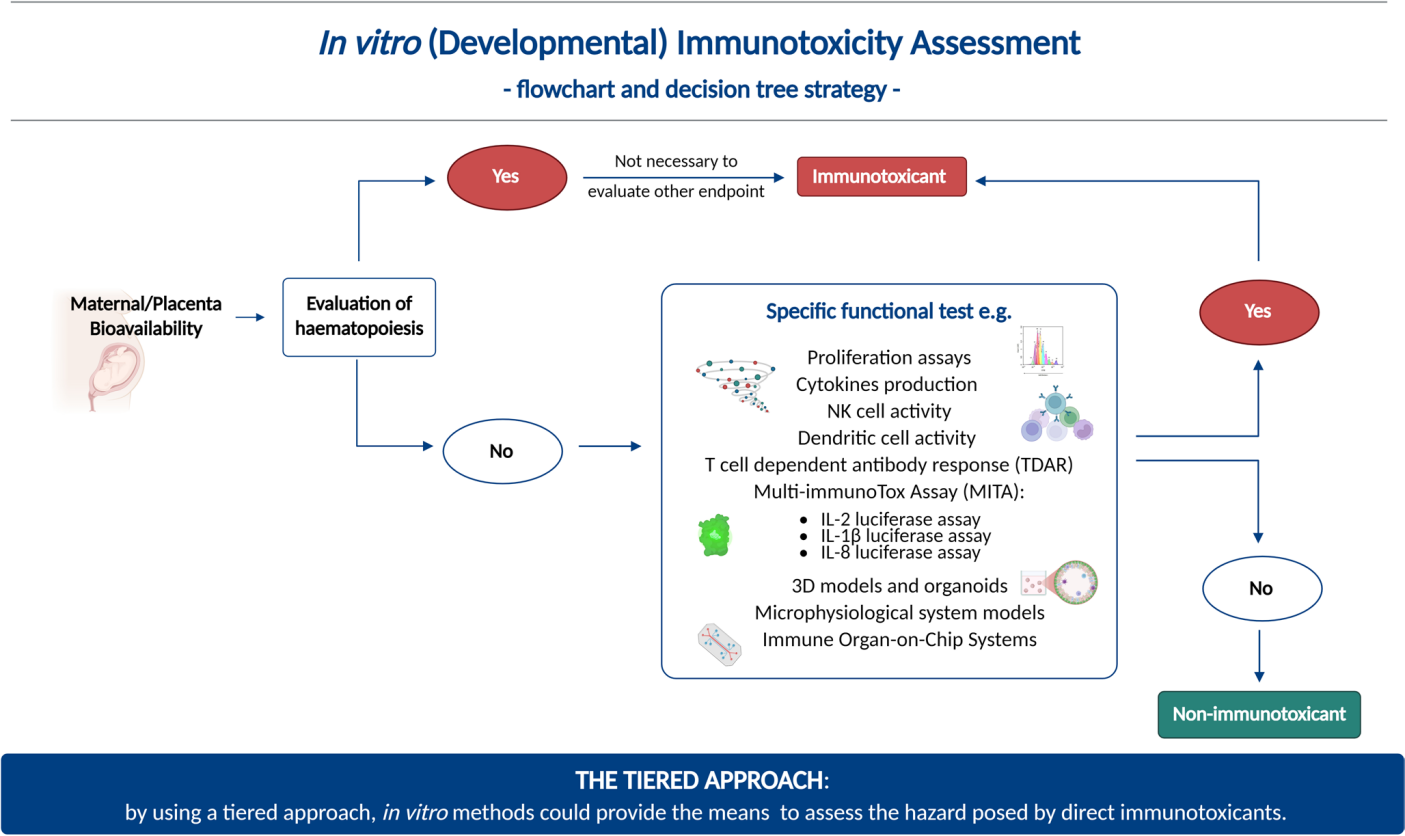
Tiered in vitro strategy for DIT assessment. The flowchart illustrates a decision-tree approach starting from maternal–placental bioavailability and evaluation of hematopoiesis, followed by targeted functional immune assays where required (the list is not exhaustive). Based on the outcomes, substances can be classified as immunotoxic or non-immunotoxic. Created in BioRender. Iulini, M. (2026) https://BioRender.com/ioo55jm

Given the complexity of immune development, a single test remains unlikely. Instead, NAMs are increasingly being integrated into tiered testing strategies, which begin with in vitro and in silico approaches and progress toward more refined assessments. Physiological maps and AOPs provide a mechanistic basis for the selection of NAMs to be included in a test battery. These frameworks aim to capture both immunosuppressive effects and inappropriate immune activation, such as skin sensitization. Several methods have been included in OECD TGs, including the IL-2 Luc and IL-2 Luc LTT assays (TG 444A), which are designed to detect immunosuppressive activity in vitro, although their applicability for DIT remains under discussion (Johnson et al. 2025; OECD 2022). Importantly, the OECD Detailed Review Paper on in vitro immunotoxicity testing (OECD 2022) emphasizes the need for a battery of complementary assays, rather than reliance on a single endpoint. This multi-assay approach enhances the robustness of immunotoxicity evaluations and supports a more human-relevant risk assessment, especially in the context of developmental exposures. A schematic overview of a tiered in vitro strategy for DIT assessment is shown in Fig. 3.

Before initiating any DIT in vitro studies, it is essential to first consider maternal systemic exposure and the potential for placental transfer, as these factors determine whether the developing fetus is likely to encounter the substance of interest. A thorough assessment of maternal toxicokinetics, chemical bioavailability, and placental transport mechanisms provides the foundation for establishing fetal exposure risk. Such preliminary evaluations ensure that subsequent in vitro investigations are biologically relevant and aligned with realistic exposure scenarios. Although a detailed discussion is beyond the scope of this review, it is important to acknowledge the availability of placental models with varying levels of complexity that can help determine whether a substance can cross the placenta and reaching the fetus. These include simple transwell-based trophoblast assays, ex vivo perfused placentas, and more advanced microphysiological systems (MPS) that mimic key structural and functional features of the maternal–fetal interface. Such models provide valuable tools for assessing fetal exposure potential and can complement DIT-focused in vitro studies (Cherubini et al. 2021; Martin et al. 2025).

Central for proper development of the immune system is the differentiation and maturation of immune cells. As described in the previous sections, a tightly regulated, stepwise process begins with HSCs, which migrate to distinct lymphoid organs where they undergo differentiation and specialization. With the current models, the timing of the process involving successive transitions between hematopoietic compartments, the migration of cells to primary and secondary lymphoid organs, their differentiation within these sites, and eventual maturation into fully immune competent cells has not yet been recapitulated in vitro.

### Zebrafish

One possibility in this regard is to use vertebrates at the earliest stages of differentiation when in line with established ethical guidelines and regulatory frameworks, as an alternative model strategy. For example, up to day 5 post-fertilization, zebrafish *(Danio rerio*) are in embryonic or larval stage, and within this time period, they can be used to investigate effects on innate immunity. This approach aligns with the principles of the 3Rs and provides a replacement alongside traditional in vitro and mammalian systems (Bailone et al. 2020). Recent advances in zebrafish embryo and larval assays have provided powerful tools for studying immunotoxicity, particularly effects on innate immunity, which includes whole-organism level physiology (Franza et al. 2024). During the embryonic stage the zebrafish innate immune system is functional, while the adaptive immune system is still maturing and becomes functional around 4—6 weeks post-fertilization (Novoa & Figueras 2012). Whereas mammals, several vertebrates, and some fish possess organized lymphoid tissue, this is not the case in zebrafish. Zebrafish have cellular components for the adaptive immune system but lack the structures that facilitate antigen presentation and interactions between immune cells. Hematopoiesis in zebrafish proceeds through two sequential phases: (i) primitive hematopoiesis, starting at 11 h post-fertilization, which occurs during early embryonic development and generates erythroid and myeloid progenitors; and (ii) definitive hematopoiesis, starting at 30 h post-fertilization, which leads to the formation of HSPCs that are responsible for the production of all adult blood cell lineages (Franza et al. 2024). During the period of up to day 5 post-fertilization, the zebrafish model relies fully on the innate immune response.

In fish, the medaka extended one-generation reproduction test (MEOGRT; OECD TG 240) is already established as the aquatic counterpart to the mammalian EOGRTS (OECD 2023). Building on this framework, the zebrafish extended one-generation reproduction test (ZEOGRT) is under validation, with the goal of providing a complementary model that benefits from the zebrafish’s extensive genetic resources and imaging capabilities (Cahill et al. 2024). Due to the lack of a gene locus defining the genetic sex, genetic sex determination was excluded from ZEOGRT procedures. While the ZEOGRT effectively captures early-life, reproductive, and innate immune endpoints, adaptive immune responses are less accessible because they develop later in zebrafish, which may limit the assessment of toxicant effects on adaptive immunity. A set of complementary NAMs should be used to test for effects on the adaptive immune system.

### In vitro models to study the adaptive immune system

The adaptive immune system, including T- and B-cells, matures later, around 4—6 weeks post-fertilization. It is currently more feasible to study the maturation of the different lineages of immune cells starting with human cord blood CD34 + cells. In this regard, many promising protocols have been proposed. However, none has yet been validated for regulatory use:

- differentiation of DCs and monocytes derived from iPSCs (Park et al. 2024; Senju et al. 2011)
- differentiation of B-cells from human umbilical cord CD34 + cells (Rawlings et al. 1997)
- differentiation of T-cells from human umbilical cord CD34 + cells (Trotman-Grant et al. 2021)

Taking this knowledge as a start, Khan et al. (2023) developed an in vitro stromal cell-free model that allows concurrent study of multiple human hematopoietic lineages (monocytes, neutrophils, megakaryocytes, and B-cells). The authors demonstrated that TCDD exposure drives HSPC to differentiate toward granulocytes and monocytes and suppresses lymphoid and megakaryocyte lineages.

### Microphysiological systems

Beyond these protocols for immune cell differentiation, several advanced systems have also been developed to better capture the complexity of immune ontogeny and its perturbation by xenobiotics. MPS are advanced in vitro platforms designed to reproduce aspects of human organ physiology and function in a controlled, dynamic environment. Unlike traditional assays, MPS aim to integrate structural, biochemical, and mechanical cues, thereby allowing a more faithful simulation of complex processes such as immune cell differentiation, migration, and communication (Kwee et al. 2023; Staumont et al. 2025). Here, we use the term microphysiological systems (MPS) in a broad sense, encompassing a spectrum of approaches such as three-dimensional (3D) organoids, dynamic culture platforms, and multi-organ setups. Within this category, organ-on-chip refers more specifically to micro-engineered devices that reproduce the architecture and function of distinct immune tissues under controlled microfluidic conditions, whereas organoids represent self-organizing 3D structures derived from stem cells that recapitulate aspects of tissue development and function.

In the context of DIT, MPS are particularly promising because they can model critical windows of immune maturation that are not accessible in vivo (in humans) and are poorly recapitulated by animal studies. By incorporating primary or stem cell–derived progenitors, these systems provide opportunities to investigate how xenobiotic exposures perturb lineage commitment, tolerance induction, or immune–tissue crosstalk during development (Johnson et al. 2025). While immune-focused organ-on-chip devices and 3D organoid models will be discussed in detail in the following sections, it is important to recognize that they all fall within the broader category of MPS. Together, these technologies represent a cornerstone of the paradigm shift toward human-relevant, mechanistically anchored, and ethically sustainable strategies for DIT testing (Nandre & Terse 2025; Rupar et al. 2024).

### Immune organ-on-chip systems

Immune organ-on-chip platforms have emerged as important tools for studying human immunology under controlled, physiologically relevant conditions, particularly in the context of drug discovery and development. These micro-engineered systems typically replicate individual immune environments, such as LN-, BM-, skin-, thymus-, spleen-, or tonsil-on—a-chip models. While significant progress has been made in developing single immune organ chips, replicating the complex crosstalk between immune organs remains a major technical hurdle (Li et al. 2023; Ramadan et al. 2023). There is growing interest in using these platforms to model immune hypersensitivity and inflammation. In this context, cancer immunotherapy represents a relevant application, as these immune processes play a central role in both therapeutic efficacy and immune-related adverse effects. In particular, the incorporation of secondary lymphoid organs into MPS enables the study of coordinated immune cell activation, expansion, and effector functions in response to tumor antigens. Such models could significantly enhance the design of effective immunotherapies. Immune organ-on-chip systems offer the potential to predict immunotoxicity of therapeutic candidates using human-relevant data, an essential step toward successful clinical translation and regulatory approval. However, several technical and biological challenges remain (Nandre & Terse 2025; Rupar et al. 2024; Shanti et al. 2021; Wang et al. 2023), including:

- scaling organ size appropriately;
- selecting optimal cell numbers and types;
- managing co-culture complexity;
- and design suitable perfusion systems.

Overcoming these challenges will be key to fully realizing the potential of immune-organ-on-chip technologies in biomedical research, therapeutic development, and immunotoxicity. Studying DIT adds an additional layer of complexity, given that the model needs to account for the extensive changes in the immune system during several developmental windows. Organ-on-chip models offer new opportunities to include, *e.g.*, the interplay between various organs, but the applicability of these models to mimic the complex and dynamic interplay involved in DIT remains to be explored.

### 3D models and organoids

Due to the crucial role of the thymus in T-cell maturation and immune tolerance, in vitro thymus models are highly relevant to DIT. Two-dimensional thymic cultures offer limited support for thymopoiesis, whereas 3D stromal cultures better promote immature T-cell development, as they more closely reproduce the spatial organization, cell–cell contacts, and microenvironmental cues required for thymocyte selection and maturation. The 3D model preserves the natural cell morphology and enables heterogeneous interactions between the cell surface and surrounding medium, promoting enhanced cell viability and proliferation. Thymic organoids have emerged as powerful tools for recapitulating key aspects of thymus physiology and function (Hübscher et al. 2024). To more accurately study thymic T-cell maturation, several advanced in vitro systems have been developed. Among them, fetal thymus organ cultures are well-established models that replicate key aspects of thymic T-cell development, including positive and negative selection and the establishment of T-cell tolerance through interactions with self-major histocompatibility complex molecules and endogenous antigens. These models have been successfully established across various platforms, including OP9-DL1/DL4 and MS5-DL1/DL4 feeder cell systems, which support de novo T-cell development from stem cells. Additionally, re-aggregated thymus organ cultures and fetal thymus organ cultures have been instrumental in modeling early thymic organogenesis. Tissue-engineering approaches using hydrogels or native scaffolds have further advanced the field by enabling in vivo reproduction of thymic function, offering promising applications for regenerative medicine (Pala et al. 2024). These systems provide valuable insights into early immune development and help reduce reliance on animal models in immunotoxicity research (Anderson et al. 2007). Recently, a miniaturized approach for thymus organoid production has been established from murine fetal thymus cells that facilitates T-cell commitment and development, replicates essential structural and functional features of the native thymus, and is suitable for live imaging as well as medium- to high-throughput applications (Major et al. 2025). Although these models have been invaluable for elucidating the signaling pathways and cellular mechanisms underlying T-cell development, accurately replicating the complex lymph stromal interactions across the diverse functional niches of the thymus remains a significant challenge. None has so far been used to assess in vitro DIT. A promising adaptation of these models is to incorporate human thymic epithelial cells and stromal cells, whether primary or derived from iPSCs. Such models will enable the modeling of distinct functional niches within the human thymus. These advanced models can be leveraged to screen thymus toxicants, opening new avenues for in vitro DIT.

In parallel, advanced human in vitro immune models are emerging as valuable tools for functional immunotoxicity assessment. Tonsil-derived models contain a broad spectrum of immune cell populations, including B-cells (naïve, memory, pre-germinal center, germinal center, and plasmablasts), T-cells (naïve, central memory, and effector memory CD4⁺ and CD8⁺ subsets), and monocytes. These models are capable of mounting adaptive immune responses following exposure to vaccines (Bonaiti et al. 2024; Kastenschmidt et al. 2023). Although tonsil-derived systems are not designed to assess DIT, they can be highly effective for evaluating immunotoxic effects in children and adults because they provide accurate, human-relevant immune responses.

To conclude this paragraph, CD34 + cell-based assays, MPS models, and organoids represent promising tools for evaluating DIT. However, to fully realize their potential, further optimization and standardization are needed. At present, these models vary in performance, and consistent protocols have yet to be established across laboratories. Additionally, the limited number of chemicals tested so far highlights the need to expand the dataset, particularly through the identification and use of well-characterized reference compounds that can serve as benchmarks for validation. Moreover, there is a need to broaden the range of functional immune endpoints assessed. Future studies should explore markers of immune activation, cytokine release profiles, and immunoglobulin production to better capture the complexity of immune responses. Integrating such functional readouts will enhance the biological relevance of these models and improve their utility in assessing both immunosuppressive and immunostimulatory effects of xenobiotics. Further progress in tissue engineering and bioengineering would enable improved 3D cell culture techniques to efficiently predict immunotoxicity, possibly including DIT.

### From exposure to outcome: epidemiology and high-dimensional immune profiling

Human epidemiological studies play a critical role in immunotoxicology by providing evidence at relevant exposure levels and in the most relevant species, humans. Associations between chemical exposures and functional immune endpoints, such as reduced vaccine responses following PFASs exposure, have been instrumental in demonstrating real-world relevance and informing regulatory risk assessment, as exemplified by their use in European Food Safety Authority (EFSA) evaluations. At the same time, epidemiological data are inherently limited by their observational nature, potential confounding factors, and the difficulty of establishing causality, highlighting the need for complementary mechanistic approaches.

In this context, it is important to differentiate between observational immune measures (e.g. immune cell frequencies or basal biomarker levels) and functional immune endpoints that directly assess immune competence, such as antigen-specific responses to vaccination or infection. Functional immune tests are widely regarded as more sensitive and biologically relevant indicators of immunotoxic effects, as they encompass the integrated performance of the immune system and are more closely associated with adverse health outcomes. Considering this, deep immune profiling and systems immunology approaches have recently emerged as powerful tools in human immunotoxicology (Nygaard et al. 2021; Ronsmans et al. 2022; Tursi et al. 2024). High-dimensional immune profiling captures the complexity of the immune system by simultaneously assessing multiple cell populations, activation states, and functional markers, thereby enabling the identification of effect biomarkers in the form of immune cell signatures or altered subpopulations. Beyond descriptive associations, these approaches provide mechanistic insight into immune pathways and responses perturbed by exposures to immunotoxic compounds.

The integration of high-dimensional immune profiling with epidemiological data and targeted in vitro experimental studies has the potential to bridge exposure–outcome associations with underlying cellular and molecular mechanisms at relevant exposure levels. By examining overlaps between immune profiles associated with developmental exposures and adverse health outcomes, such as transcriptomic signatures in cord blood linked to prenatal PFASs exposure and impaired immune function in early childhood (Pennings et al. 2016), the evidence for causal links can be strengthened. Importantly, the identification of sensitive and robust immune cell signatures reflecting immunomodulatory effects can inform the development of future in vitro screening assays for DIT and support the identification of key events for AOP development.

## Challenges in advancing DIT testing

The immune system plays a central role in shaping a wide range of health outcomes throughout life. Despite major progress in understanding immune system ontogeny and in developing human-relevant NAMs, several challenges still limit the implementation of DIT testing in regulatory frameworks. These challenges fall into two main categories: knowledge gaps and methodological gaps, which together hinder the development, standardization, and validation of predictive testing strategies.

### Knowledge gaps limiting DIT assessment

A major gap in our current understanding of DIT is the limited knowledge of which specific immune developmental processes and windows of susceptibility are most affected by developmental immunotoxicants, and through which mechanisms. This uncertainty is closely linked to the limited availability of human reference data describing normal immune development across prenatal and early postnatal life. Compared with rodent models, human datasets remain sparse, fragmented, and often derived from small cohorts or opportunistic sampling. As a result, it is difficult to (i) define baseline trajectories of immune maturation, (ii) identify critical windows of susceptibility, (iii) map cellular and molecular events essential for immune competence and (iv) distinguish normal developmental variability from toxicant‑induced alterations.

Recent single‑cell maps have begun to fill some of these gaps, but they still cover only selected tissues and developmental windows. Moreover, the integration of multi‑omics datasets into coherent mechanistic frameworks remains incomplete. Consequently, many NAMs lack the developmental context needed to ensure that they capture biologically relevant processes. This uncertainty directly impacts the selection and development of in vitro DIT assays, as it remains unclear which key events should be prioritized. Addressing these knowledge gaps requires a more comprehensive and human‑relevant understanding of immune system development, which can subsequently be translated into feasible, mechanistically anchored, and reliable DIT testing strategies.

### Emerging technologies for immune system mapping

To address these knowledge gaps, several emerging technologies now offer unprecedented resolution in mapping immune development and function. Emerging single-cell technologies enable unprecedented mapping of the human immune system across developmental stages and functional states. scRNA-seq of multiple organs of the developing human immune system (He et al. 2025; Suo et al. 2022) provides a systematic and comprehensive framework to describe immune cell differentiation and maturation. To specifically address lymphocytes development and clonal dynamics, scRNA-seq can be combined with single cell TCR or antibody sequencing. Such high-resolution developmental maps provide a critical foundation for the design of NAMs capable of detecting adverse effects targeting specific key events or sensitive developmental windows.

Beyond developmental mapping, single-cell high-dimensional functional immune profiling and systems immunology approaches in human studies provide powerful tools to identify biomarkers of effect (Nygaard et al. 2021; Ronsmans et al. 2022; Tursi et al. 2024). These approaches offer an important link between exposure and immune outcomes in humans, enabling the detection of DIT effects under real-life exposure conditions and supporting the biological anchoring of effects observed in NAM-based testing strategies.

### Future perspectives in DIT assessment

Looking ahead, progress in (developmental) immunotoxicology will depend on the generation and integration of larger, high-quality datasets capable of distinguishing between biomarkers of broad immunosuppression and those that are specific to individual chemical classes. Such differentiation is critical for improving the precision of hazard identification and establishing mechanistic links between exposure and immune dysfunction. Expanding the repertoire of immune cell targets, including both circulating and tissue-resident populations, will further enhance the sensitivity and specificity of (developmental) immunotoxicity assessments.

Advances in in vitro models are poised to play a central role in this evolution. Emerging systems (including 2D and 3D cultures, MPS, and co-culture platforms) offer improved opportunities to study indirect immunotoxic effects by capturing dynamic interactions between immune cells, stromal cells, and barrier tissues. Coupled with multiomics approaches, these models are expected to deepen our mechanistic understanding of chemical-induced immune alterations and support the identification of more robust and human-relevant toxicological endpoints. Yet, in vitro models for adult immunotoxicity are not always able to capture the different windows of susceptibility in the developing immune system needed for DIT testing.

While several NAMs for DIT have been published, only a few have progressed toward validation and regulatory acceptance. One important barrier to regulatory implementation is the limited availability of human-relevant reference data that can be used to define adversity and benchmark in vitro findings across different developmental stages of the immune system. In addition, validation of NAMs for DIT remains challenging due to the complexity of immune ontogeny, the life-stage specificity of immune processes, and the lack of established gold-standard assays for many developmental immune endpoints. These limitations complicate the establishment of performance standards and the demonstration of predictive capacity required for regulatory acceptance. As a result, significant efforts are needed to translate mechanistic insights and NAM-derived endpoints into formats compatible with regulatory decision-making. Regulatory use of emerging in vitro models will require clearly defined contexts of use and the establishment of appropriate performance standards.

Harmonization and standardization are crucial to ensure reproducibility and robustness. Standardization of protocols, culture conditions, and reporting practices will improve inter-laboratory reproducibility and facilitate transferability between laboratories. These improvements will also enhance the feasibility of large-scale initiatives aimed at identifying immunotoxic hazards earlier in the chemical evaluation pipeline. These efforts will require international coordination of round robin studies. IATAs and defined approaches are expected to play a key role in enabling the regulatory implementation of NAMs for DIT. Because DIT involves multiple developmental processes and immune compartments, it is unlikely that a single assay will be sufficient to predict adverse outcomes. Instead, combinations of complementary NAMs capturing different key events across immune development will likely be required. Establishing internationally agreed test batteries, together with transparent data interpretation procedures and performance criteria, will therefore be essential for supporting regulatory acceptance.

Finally, sustained international coordination will be essential to translating scientific advancements into regulatory practice. Collaboration among academia, regulatory agencies, and international organizations such as the OECD will be crucial to achieve this goal. Obtaining global consensus on testing strategies, performance criteria, and data interpretation frameworks will support the development of validated test batteries and promote their acceptance within risk assessment frameworks. Collectively, these efforts will help build a more predictive, and mechanistically anchored human-relevant framework for DIT assessment, supporting the protection of future generations from the health consequences of exposures to (developmental) immunotoxicants.

## Data Availability

This article is a review of previously published studies, and no new data were generated.

## Abbreviations

AGM: Aorta–gonad–mesonephros
AOP: Adverse outcome pathway
BM: Bone marrow
BPA: Bisphenol A
DCs: Dendritic cells
DIT: Developmental immunotoxicity
ECHA: European chemicals agency
EFSA: European food safety authority
EGFP: Enhanced green fluorescent protein
EMA: European medicines agency
EOGRTS: Extended one-generation reproductive toxicity study
GFP: Green fluorescent protein
HIDmap: Human Immune System Development Map
HSCs: Hematopoietic stem cells
HSPC: Hematopoietic stem-progenitor cell
IFN: Interferon
IL: Interleukin
ILC: Innate lymphoid cell
iPSCs: Induced pluripotent stem cells
IATA: Integrated approaches to testing and assessment
LNs: Lymph nodes
MCs: Mast cells
MEOGRT: Medaka extended one-generation reproduction test
MINERVA: Molecular Interaction Network Visualization platform
MPS: Microphysiological system
NAMs: New approach methodologies
NK: Natural killer (cell)
OECD: Organisation for economic co-operation and DevelopmentPARC: Partnership for the assessment of risk from chemicals
PFASs: Per- and polyfluoroalkyl substances
PFOS: Perfluorooctane sulfonate
PFOA: Perfluorooctanoic acid
PM: Particulate matter
PM 2.5: Particulate matter ≤ 2.5 µm
PM 0.1: Particulate matter ≤ 0.1 µm
scRNA-seq: Single-cell RNA sequencing
TDAR: T-cell-dependent antibody response
TG: Test guideline
TLR: Toll-like receptor
TNF-α: Tumor necrosis factor alpha
TCR: T-cell receptor
Th: T helper (cell)
Treg: Regulatory T cell
VPA: Valproic acid
YS: Yolk sac
ZEOGRT: Zebrafish extended one-generation reproduction test
