# Correction: From animal models to NAMs: a paradigm shift in developmental immunotoxicity testing

**DOI:** 10.1007/s00204-026-04476-y

**Published:** 2026-06-15

**Authors:** Martina Iulini, Véronique de Bruijn, Selma Hurem, Unni C. Nygaard, Saadia Kerdine-Römer, Christiane Spruck, Julia Tigges, Rob Vandebriel, Emanuela Corsini

**Affiliations:** 1 Laboratory of Toxicology and Risk Assessment, Department of Pharmacological and Biomolecular Sciences ‘Rodolfo Paoletti’, Università Degli Studi Di Milano, Via Balzaretti 9, 20133 Milan, Italy; 2 Centre for Health Protection, National Institute of Public Health and the Environment (RIVM), Bilthoven, The Netherlands; 3 Faculty of Veterinary Medicine, Norwegian University of Life Sciences (NMBU), Ås, Norway; 4 Section for Immunology, Norwegian Institute of Public Health, Oslo, Norway; 5 Faculty of Pharmacy, Université Paris-Saclay, Paris, France; 6 Düsseldorf, Germany

**Correction to: Archives of Toxicology** **https://doi.org/10.1007/s00204-026-04424-w**

**Correction for Editor Evaluation**

The authors regret to inform that several minor issues were identified in the published version of the manuscript. These issues were editorial in nature and do not affect the scientific content, results, or conclusions of the work.

**1. Correction to the Funding Section**

**Location of the error:** Funding section of the published manuscript.

**Reason for the error:** The Funding section was incomplete due to an oversight during manuscript preparation, resulting in incomplete acknowledgment of the funding sources.

**Corrected text:** The Dutch Ministry of Health, Welfare, and Sport, the Norwegian Institute of Public Health, and the European Partnership for the Assessment of Risks from Chemicals (PARC) that has received funding from the European Union’s Horizon Europe Research and Innovation Programme under Grant Agreement No. 101057014.

**Impact on the manuscript:** This correction does not affect the results or conclusions of the manuscript.

**2. Correction of Typographical Error**

**Location of the error:** Author contribution statement.

**Reason for the error:** A typographical error was introduced during manuscript preparation.

**Table:** 

Published version	Corrected version
“Martina Iulini and Véronique de Bruijy have contributed eqaully to this work”	“Martina Iulini and Véronique de Bruijn have contributed equally to this work”

**Impact on the manuscript:** This correction does not affect the results or conclusions of the manuscript.

**3. Formatting Correction in the Abbreviation List**

**Location of the error:** Abbreviation list.

**Reason for the error:** A formatting issue caused the abbreviation “PARC” not to appear on a separate line.

**Corrected version:** The abbreviation “PARC” has been moved to a new line in the abbreviation list.

**Impact on the manuscript:** This correction does not affect the results or conclusions of the manuscript.

**4. Formatting Correction in the Data Availability Section**

**Location of the error:** PDF version of the manuscript, “Data availability” section.

**Reason for the error:** A formatting inconsistency occurred during PDF generation, resulting in a different font appearance in the “Data availability” section.

**Corrected version:** The font formatting of the “Data availability” section has been corrected to match the rest of the document.

**Impact on the manuscript:** This correction does not affect the results or conclusions of the manuscript.

**Author Statement**

The authors apologize for any inconvenience caused by these errors and confirm that the scientific results and conclusions of the manuscript remain unchanged.

